# Eprenetapopt triggers ferroptosis, inhibits NFS1 cysteine desulfurase, and synergizes with serine and glycine dietary restriction

**DOI:** 10.1126/sciadv.abm9427

**Published:** 2022-09-14

**Authors:** Kenji M. Fujihara, Bonnie Z. Zhang, Thomas D. Jackson, Moses O. Ogunkola, Brunda Nijagal, Julia V. Milne, David A. Sallman, Ching-Seng Ang, Iva Nikolic, Conor J. Kearney, Simon J. Hogg, Carlos S. Cabalag, Vivien R. Sutton, Sally Watt, Asuka T. Fujihara, Joseph A. Trapani, Kaylene J. Simpson, Diana Stojanovski, Silke Leimkühler, Sue Haupt, Wayne A. Phillips, Nicholas J. Clemons

**Affiliations:** ^1^ Gastrointestinal Cancer Program, Cancer Research Division, Peter MacCallum Cancer Centre, Melbourne, Victoria, Australia.; ^2^ Sir Peter MacCallum Department of Oncology, The University of Melbourne, Parkville, Victoria, Australia.; ^3^ Department of Biochemistry and Pharmacology, The University of Melbourne, Parkville, Victoria, Australia.; ^4^ Bio21 Molecular Science and Biotechnology Institute, The University of Melbourne, Parkville, Victoria, Australia.; ^5^ Institute of Biochemistry and Biology Department for Molecular Enzymology, University of Potsdam, Potsdam, Germany.; ^6^ Metabolomics Australia, The Bio21 Institute of Molecular Science and Biotechnology, The University of Melbourne, Parkville, Victoria, Australia.; ^7^ Malignant Hematology Department, H. Lee Moffitt Cancer Center and Research Institute, Tampa, FL, USA.; ^8^ Victorian Centre for Functional Genomics, Peter MacCallum Cancer Centre, Melbourne, Victoria, Australia.; ^9^ Translational Hematology Program, Cancer Research Division, Peter MacCallum Cancer Centre, Melbourne, Victoria, Australia.; ^10^ Human Oncology and Pathogenesis Program, Memorial Sloan Kettering Cancer Center, New York, NY, USA.; ^11^ Department of Surgical Oncology, Peter MacCallum Cancer Centre, Melbourne, Victoria, Australia.; ^12^ Cancer Immunology Program, Peter MacCallum Cancer Centre, Melbourne, Victoria, Australia.; ^13^ Tumor Suppression and Cancer Sex Disparity Laboratory, Peter MacCallum Cancer Centre, Melbourne, Victoria, Australia.; ^14^ Department of Surgery (St. Vincent’s Hospital), The University of Melbourne, Parkville, Victoria, Australia.; ^15^ Department of Biochemistry and Molecular Biology, Monash University, Clayton, Victoria, Australia.

## Abstract

The mechanism of action of eprenetapopt (APR-246, PRIMA-1^MET^) as an anticancer agent remains unresolved, although the clinical development of eprenetapopt focuses on its reported mechanism of action as a mutant-p53 reactivator. Using unbiased approaches, this study demonstrates that eprenetapopt depletes cellular antioxidant glutathione levels by increasing its turnover, triggering a nonapoptotic, iron-dependent form of cell death known as ferroptosis. Deficiency in genes responsible for supplying cancer cells with the substrates for de novo glutathione synthesis (*SLC7A11*, *SHMT2*, and *MTHFD1L*), as well as the enzymes required to synthesize glutathione (*GCLC* and *GCLM*), augments the activity of eprenetapopt. Eprenetapopt also inhibits iron-sulfur cluster biogenesis by limiting the cysteine desulfurase activity of NFS1, which potentiates ferroptosis and may restrict cellular proliferation. The combination of eprenetapopt with dietary serine and glycine restriction synergizes to inhibit esophageal xenograft tumor growth. These findings reframe the canonical view of eprenetapopt from a mutant-p53 reactivator to a ferroptosis inducer.

## Teaser

The mutant-p53 reactivator, eprenetapopt, kills tumor cells through inducing ferroptosis and not apoptosis.

## INTRODUCTION

*TP53* mutation occurs in ~50% of all cancers, and overall is associated with poor survival (*1*), providing a strong impetus for the development of mutant-p53 (mut-p53) targeted therapeutics. Eprenetapopt (APR-246, PRIMA-1^MET^) was developed as a mut-p53 targeted therapy and is currently under clinical investigation in *TP53*-mutated myelodysplastic syndromes (MDS) and acute myeloid leukemia (AML) (*2*). Recent results from two phase 2 clinical trials in *TP53-*mutated MDS, combining eprenetapopt with standard-of-care azacitidine, reported rates of complete remission (CR) of ~50% (*3*, *4*). Furthermore, apparent improvements in median overall survival compared to previous studies of azacitidine alone were reported in *TP53*-mutated MDS (*3*, *4*). However, early results from the phase 3 randomized clinical trial in *TP53*-mutated MDS failed to meet the primary end point of CR rate (33.3% in eprenetapopt + azacitidine, 22.4% in azacitidine alone, *P* = 0.13; www.aprea.com).

The therapeutic effect of eprenetapopt has been understood to involve the reactivation of wild-type p53 activity to induce apoptosis through covalent modification of cysteine residues in the core domain of mut-p53 protein (*5*). The specific mechanism of action (MoA) of eprenetapopt, however, remains to be thoroughly defined. Recently, we demonstrated that the expression of *SLC7A11*, which encodes the functional subunit of the cystine-glutamate antiporter, system x_c_^−^, is a superior determinant of response to eprenetapopt across cancer lineages, surpassing both *TP53* mutation status and p53 protein levels (*6*). The import of cystine through system x_c_^−^ provides the predominant source of intracellular cysteine, which is the rate-limiting substrate required for de novo synthesis of the cellular antioxidant glutathione (GSH). Eprenetapopt and its structural analog APR-017 (also known as PRIMA-1) are prodrugs that undergo spontaneous conversion to the active compound, 2-methylene 3-quinuclidinone (MQ) (*5*). MQ is a thiol-reactive Michael acceptor that forms reversible covalent bonds with thiol-containing molecules including cysteine and the GSH (*7*, *8*). As a result, the anticancer effects of eprenetapopt have been linked to GSH depletion and inhibition of the thioredoxin antioxidant system (*7*–*9*). Notably, we previously demonstrated that mut-p53 protein accumulation drives down the expression of *SLC7A11* through the inhibition of the transcription factor NRF2 (nuclear factor erythroid 2-related factor 2, encoded by *NFE2L2*), providing an explanation for the selectivity of eprenetapopt against mut-p53 cancer cells under some conditions (*8*). However, to date, limited unbiased examinations of the MoA of eprenetapopt have been attempted, and the predominant focus of prior investigations has been through the lens of eprenetapopt as a mut-p53 reactivator a priori.

In this study, we undertook a systematic, unbiased approach to probe the MoA of eprenetapopt, incorporating genome-wide CRISPR perturbation screening and metabolite and proteomic profiling in cancer cells with pan-cancer gene dependency and cell line sensitivity datasets. In this way, we provide a compendium of functional genomic and molecular detail into the MoA of eprenetapopt. In particular, CRISPR perturbation screens reveal that loss of mitochondrial one-carbon (mito-1C) metabolism enzymes, mitochondrial serine hydroxymethyltransferase 2 (SMHT2) and methylenetetrahydrofolate dehydrogenase 1-like (MTHFD1L), sensitizes cancer cells to eprenetapopt—an insight that can be exploited in vivo to sensitize tumors to eprenetapopt with serine and glycine (SG) dietary restriction. Mechanistically, we find that eprenetapopt induces GSH depletion, triggering iron-dependent, nonapoptotic ferroptosis, and limits cellular proliferation, in part, by inhibiting the cysteine desulfurase activity of NFS1. Clinically, these insights may provide a rational avenue for the expansion of eprenetapopt utility in MDS to include patient selection based on the presence of ring sideroblasts, rather than reliance on *TP53* mutation status.

## RESULTS

### Multiomics strategy to determine the MoA of eprenetapopt

**Fig. 1.**
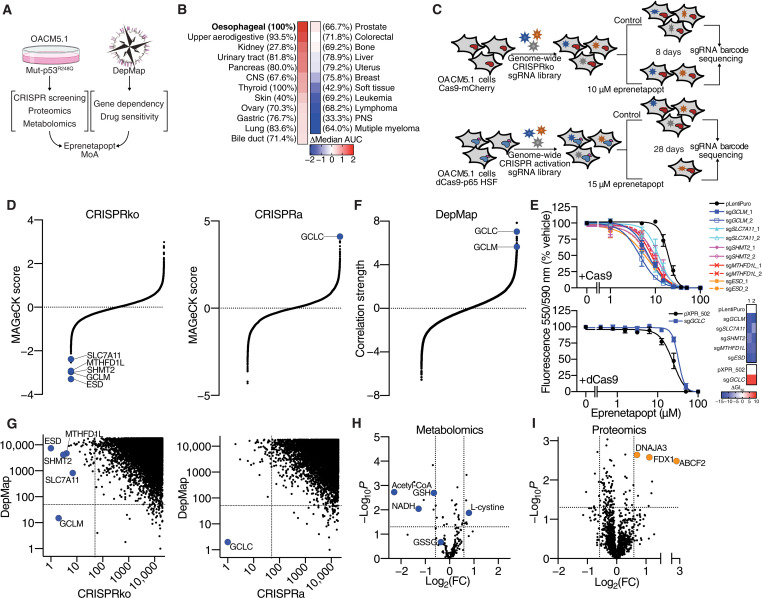
Multiomics strategy to determine the MoA of eprenetapopt. (**A**) Schematic diagram showing a strategy to determine the MoA of eprenetapopt. (**B**) Heatmap of sensitivity to eprenetapopt analog (APR-017). Cancer lineages are ordered by sensitivity determined by delta median, area under the curve (AUC) of compound activity. Percentages denote the frequency of *TP53* mutations in each lineage. CNS, central nervous system; PNS, peripheral nervous system. (**C**) Schematic showing the workflow for the CRISPRko and CRISPRa screens in OACM5.1 cells. (**D**) MAGeCK scores (negative indicating dropout and positive indicating “enrichment”) from CRISPR screens, plotted in order of magnitude. (**E**) Cellular metabolic activity measured by alamarBlue as a surrogate readout for cell viability following 72-hour exposure with eprenetapopt at indicated doses in cells transduced with individual sgRNA targeting identified hits. Heatmap represents the change in GI_50_ (dose where 50% growth inhibition is achieved) relative to control. (**F**) Fisher’s transformed *z*-scored Pearson correlation strength of gene dependency from the DepMap database with eprenetapopt analog (APR-017) sensitivity data from CTRPv2, plotted in order of magnitude. (**G**) Comparison of CRISPR screens and DepMap gene dependency data shows overlay of glutamate-cysteine ligase units (GCLC and GCLM). Plot is representing the rank of top hits (ordered by dropout in CRISPRko, by enrichment in CRISPRa, and by positive correlations in DepMap). Dashed lines indicate overlap of top 50 ranked genes. (**H**) Changes in polar metabolites determined by untargeted liquid chromatography–mass spectrometry (LC-MS) metabolomics and (**I**) proteins determined by label-free quantitative proteomics in OACM5.1 cells following treatment with 50 μM eprenetapopt for 12 hours compared to vehicle. Dotted lines indicate significance cutoffs {*P* < 0.05, |log_2_[fold change (FC)]| > 0.5}. Two-tailed unpaired *t* test (H and I). Error bars = SEM. (D) *n* = 2 for CRISPRko, *n = 1* for CRISPRa, (E) *n* = 3 to 4, (H) *n* = 6, and (I) *n* = 4. See also fig. S1.

To determine the MoA of eprenetapopt, we undertook a set of complementary approaches, including genome-wide CRISPR perturbation screens, quantitative proteomics, and untargeted metabolomics in response to eprenetapopt (Fig. 1A). We also leveraged and incorporated publicly available pan-cancer therapeutic response and cancer cell line gene dependency datasets from the Broad Institute’s DepMap portal (www.depmap.org) to expand the validity and test the generalizability of our investigational datasets. To select an appropriate model system to systematically dissect the MoA of eprenetapopt, we first analyzed cancer cell line drug sensitivity data from the Cancer Therapeutic Response Portal v2 (CTRPv2). We found that esophageal cancer cell lines with a high rate of *TP53* mutation were the most resistant to the structural analog of eprenetapopt, APR-017 (Fig. 1B). Specifically, we selected the OACM5.1 esophageal cancer cell line for our investigation, which expresses the most common *TP53* missense mutation across all cancers, R248Q (mut-p53^R248Q^). As a result, this model is amenable to identifying genetic determinants of sensitivity to eprenetapopt without precluding mut-p53 reactivation as a MoA.

We transduced OACM5.1 cells expressing Cas9-mCherry with the Brunello genome-wide CRISPR knockout (CRISPRko) library (*10*) and challenged these cells with a sublethal dose of eprenetapopt for 8 days (Fig. 1C and fig. S1A). In parallel, we transduced OACM5.1 cells expressing dCas9-VP64 with the Calabrese Set A genome-wide CRISPR activation (CRISPRa) library (*11*) and challenged these cells with a lethal dose of eprenetapopt for 28 days. The CRISPRko screen aimed to identify genetic deletions that increase sensitivity to eprenetapopt and thus “dropout” in the eprenetapopt-treated cells compared to vehicle treated controls. Meanwhile, the CRISPRa screen aimed to identify gene overexpression that protects cells from eprenetapopt. The CRISPRa screen has the advantage of potentially identifying genetic modulators of eprenetapopt sensitivity that may not be revealed in the CRISPRko screen, where general essential genes drop out during the puromycin selection.

Consistent with our prior observations, and those of others, that eprenetapopt triggers GSH depletion in cancer cells (*7*, *8*), the CRISPRko screen identified genes involved in GSH synthesis, *SLC7A11* and *GCLM*, while *GCLC* was the top enriched gene in the CRISPRa screen (Fig. 1D). Genes encoding apoptotic machinery (including proapoptotic targets of p53), antiapoptotic genes, and *TP53* itself were not identified as modulators of eprenetapopt response in either screen, suggesting that reactivation of mut-p53 and induction of apoptosis are dispensable for eprenetapopt-mediated cell death. Notably, two mito-1C metabolism genes, *SHMT2* and *MTHFD1L*, and the *S*-formylglutathione hydrolase gene, *ESD*, involved in GSH-mediated formaldehyde detoxification (*12*) were identified in the CRISPRko screen. We confirmed that two independent single-guide RNA (sgRNA) guides targeting *GCLM*, *SLC7A11*, *SHMT2*, *MTHFD1L*, and *ESD*, as well as one sgRNA guide for *GCLC* activation, modulated sensitivity to eprenetapopt as expected (Fig. 1E). We confirmed gene disruption in a subset of our top gene candidates (fig. S1B) and overexpression of *GCLC* following guide transduction (fig. S1C). In agreement, genome-wide dependency data demonstrated that *GCLC* and *GCLM* dependency correlate strongly with pan-cancer cell line sensitivity to APR-017 (Fig. 1F and fig. S1B). *GCLC* and *GCLM* uniquely scored strongly in both our screening approaches, indicating that the modulation of the de novo GSH axis is central to the MoA of eprenetapopt in cancer cells (Fig. 1G).

Complementing our functional genomics approach, we performed untargeted metabolomics and label-free quantitative proteomics in OACM5.1 cells treated with eprenetapopt before the onset of cell death. As expected, eprenetapopt treatment decreased reduced GSH levels, without increasing oxidized GSH (GSSG) levels (Fig. 1H), indicating that eprenetapopt is triggering total GSH depletion rather than the conversion of reduced GSH to GSSG. Furthermore, cystine levels were increased following eprenetapopt treatment, which could be explained by the up-regulation of *SLC7A11* expression in response to eprenetapopt treatment shown in previous studies (*7*, *13*). Meanwhile, mitochondrial metabolism was altered, as demonstrated by the decrease in acetyl–coenzyme A (CoA) and reduced form of nicotinamide adenine dinucleotide (NADH) (Fig. 1H). Notably, our proteomics study revealed that cells treated with eprenetapopt up-regulated the levels of mitochondrial ferredoxin 1 (FDX1), a critical component of mitochondrial iron-sulfur cluster (ISC) biosynthesis (*14*), as well as two other proteins, DNAJA3 and ABCF2, which have reported roles in iron metabolism (Fig. 1I) (*15*, *16*). Together, these datasets provide confirmation that GSH depletion is central to the MoA of eprenetapopt and suggests as yet unappreciated roles for mito-1C metabolism and iron metabolism in the MoA of eprenetapopt.

### Eprenetapopt and mito-1C metabolism

**Fig. 2.**
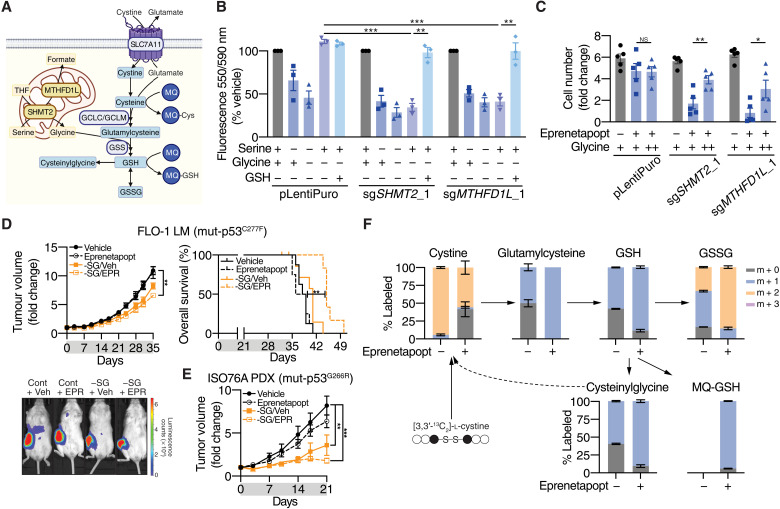
Eprenetapopt and mito-1C metabolism. (**A**) Schematic diagram illustrating the connections between de novo GSH synthesis and mito-1C metabolism. THF, tetrahydrofolate; GSH, reduced GSH; MQ-Cys, MQ-conjugated cysteine; MQ-GSH; MQ-conjugated GSH. (**B**) Cellular metabolic activity measured by alamarBlue as a surrogate readout for cell viability compared to complete medium (CM) following 72 hours of serine, glycine, or SG deprivation and glycine deprivation rescued with 1 mM GSH monoethyl ester (GSH) in OACM5.1 cells. (**C**) Relative cell number following treatment with 10 μM eprenetapopt ± 1 mM glycine supplementation for 4 days. + indicates glycine in RPMI 1640, and ++ indicates supplementation with additional 1 mM glycine. (**D**) Top: Growth curves and overall survival (time to reach a tumor volume of ≥1400 mm^3^) of nonobese diabetic–severe combined immunodeficient interleukin-2Rγ^KO^ (NSG) mice inoculated with FLO-1 LM tumors treated with eprenetapopt (EPR; 100 mg/kg, daily) or vehicle on either normal or SG-free diets for 35 days. Bottom: Representative bioluminescence images illustrating the metastatic burden in mice after 35 days of treatment. (**E**) Growth curves of esophageal adenocarcinoma PDXs in NSG mice treated with eprenetapopt (100 mg/kg, daily) or vehicle on either normal or SG-free diet for 21 days. (**F**) Fractional labeling of de novo GSH synthesis intermediates from ^13^C_2_-cystine following 12-hour exposure to 50 μM eprenetapopt compared to vehicle in OACM5.1 cells. Two-tailed *t* test (B and C), one-way analysis of variance (ANOVA) with Tukey’s multiple comparisons test (D and E), and log-rank (Mantel-Cox) test (D). *P < 0.05, **P < 0.01, and ***P < 0.001. NS, not significant. Error bars = SEM. (A) *n* = 1, (B and F) *n* = 3, (C) *n* = 5, (D) *n* = 8, and (E) *n* = 4 to 5. See also fg. S2.

We next investigated the involvement of mito-1C metabolism in eprenetapopt sensitivity. Notably, mito-1C metabolism intersects with de novo GSH synthesis through SHMT2-mediated production of glycine from serine and supports cellular proliferation through formate production (Fig. 2A) (*12*). First, we attempted to rescue the increased sensitivity to eprenetapopt in mito-1C–deficient cells using exogenous formate. Pertinently, formate supplementation did not alter sensitivity to eprenetapopt (fig. S2A). Previous studies have shown that mito-1C–deficient cells have altered dependency on exogenous glycine due to the loss of endogenous glycine production by SHMT2 (*17*). Cells expressing *SHMT2* and *MTHFD1L* sgRNAs displayed reduced growth in the absence of glycine, while cells transduced with control vectors did not (Fig. 2B and fig. S2B). Meanwhile, all cells displayed sensitivity to serine deprivation alone and combined SG deprivation. The glycine auxotrophy in mito-1C–deficient cells could be reversed with the exogenous supply of cell permeable GSH (Fig. 2B and fig. S2B), in agreement with previous studies (*17*). This highlights that mito-1C–deficient cells have a higher demand for exogenous glycine for de novo GSH synthesis. As a result, the increased sensitivity to eprenetapopt in mito-1C–deficient cells can be reversed by supplementation of additional glycine to the cell medium (Fig. 2C and fig. S2B). Together, these data demonstrate that mito-1C metabolism interplays with de novo GSH synthesis by providing endogenous glycine to regulate sensitivity to eprenetapopt in cancer cells.

Since mito-1C deficiency leads to increased demand for exogenous glycine following eprenetapopt-induced GSH depletion, we reasoned that limiting glycine availability by restricting exogenous SG would sensitize cancer cells to respond to eprenetapopt. Restriction of dietary SG has previously been shown to reduce tumor growth in a variety of xenografts and genetically engineered mouse models and demonstrated strong efficacy in combination with biguanides (*18*). To this end, we tested the combination of dietary SG restriction and eprenetapopt in an aggressive model of spontaneous metastatic esophageal cancer, FLO-1 LM (mut-p53^C277F^) (*19*). Five weeks of daily dosing of eprenetapopt (100 mg/kg), monotherapy failed to inhibit primary tumor growth or limit metastatic spread (Fig. 2D and fig. S2, D and E). Promisingly, we found that the combination of SG restriction and eprenetapopt significantly inhibited both primary tumor growth and delayed the onset of metastatic disease, leading to prolonged overall survival (Fig. 2D and fig. S2D). Dietary SG restriction in combination with eprenetapopt was well tolerated (fig. S2F), and chow consumption remained consistent across the different diet interventions (fig. S2G). We also demonstrated that the combination of eprenetapopt and SG restriction limited growth in an esophageal adenocarcinoma patient-derived xenograft (PDX; mut-p53^G266R^) tumor model (Fig. 2E). Notably, in the metastatic and PDX models, dietary SG restriction alone inhibited tumor growth to a degree (fig. S2H). Together, these results demonstrate that SG restriction can improve the efficacy of eprenetapopt and that dietary SG restriction can inhibit the growth of esophageal cancer tumors in vivo.

Given that de novo GSH synthesis pathways are central to eprenetapopt sensitivity, we hypothesized that eprenetapopt inhibits the de novo synthesis of GSH. First, we verified that eprenetapopt does not block cystine uptake into cells, while a known cystine uptake inhibitor, erastin, does (fig. S2I). Notably, we also found that mito-1C deficiency did not alter sensitivity to erastin (fig. S2J). Furthermore, in contradiction to previous investigations (*9*), we found that eprenetapopt treatment decreases the level of glutathionylation in cells (fig. S2K), indicating that the GSH depletion induced by eprenetapopt is not due to loss of free GSH to protein conjugation. We tracked the incorporation of isotopically labeled cystine into GSH and other products using liquid chromatography–mass spectrometry (LC-MS). Notably, we observed that eprenetapopt treatment increased the proportion of cystine-derived cysteine incorporated into de novo synthesis products glutamylcysteine, GSH, and GSSG (Fig. 2F). We also detected both MQ-GSH and MQ-cysteine adducts in eprenetapopt-treated cells (Fig. 2F and fig. S2, L to N). Meanwhile, eprenetapopt treatment also increased the proportion of incorporation into GSH breakdown intermediate, cysteinylglycine (Fig. 2F). Furthermore, we found that eprenetapopt increased the proportion of unlabeled cystine in cells (Fig. 2F). Given that these cells have been treated with isotopically labeled cystine, this unlabeled cystine is likely derived from the unlabeled cysteine released from degraded GSH (Fig. 2F). This provides an alternative explanation for the increase in cystine we observed in our metabolomics study (Fig. 1H). These data strongly indicate that eprenetapopt is not inhibiting de novo synthesis of GSH and instead is triggering the degradation of GSH.Together with our data that show mito-1C and de novo GSH synthesis deficiency drive increased sensitivity to eprenetapopt, this suggests that the capacity of cancer cells to regenerate de novo GSH from the available pool of GSH precursors, in particular, cysteine and glycine, determines cancer cell sensitivity to eprenetapopt. This model is in keeping with our prior work, and confirmed by others (*7*, *8*, *20*), demonstrating that inhibiting cystine uptake with erastin or sulfasalazine synergizes with eprenetapopt both in vitro and in vivo.

### Eprenetapopt triggers ferroptosis

Ferroptosis is a form of nonapoptotic cell death characterized by the induction and accumulation of toxic lipid membrane peroxidation catalyzed by iron (*21*). Experimentally, the induction of ferroptosis is verified by the restoration of cell viability by iron chelators and lipophilic antioxidants and lack of cell death rescue by pan-caspase inhibitors. In our prior work, we showed that eprenetapopt induces lipid peroxidation following GSH depletion (*8*). However, we did not confirm that eprenetapopt induces ferroptosis as iron chelation failed to rescue cell viability following 96-hour cotreatment as determined by a resazurin-based assay, which measures the reducing capacity of cells as a surrogate for cell viability (*8*). Despite this, more recent investigation into the mechanisms of ferroptosis have highlighted that the use of metabolism-based assays may obscure results. This is because ferroptosis induced by sustained cystine deprivation or erastin treatment perturbs cell proliferation beyond the induction lipid peroxidation (*22*).

**Fig. 3.**
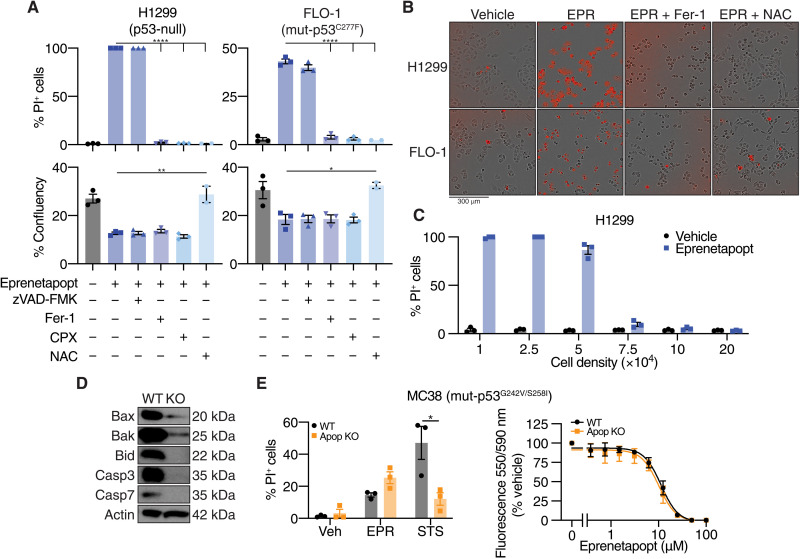
Eprenetapopt triggers ferroptosis. (**A**) Percentage (%) of dead cells [as determined by % PI^+^ cells] and percentage of cell confluency following treatment for 24 hours with 50 μM eprenetapopt with or without 50 μM zVAD-FMK (pan-caspase inhibitor), 12.5 μM Fer-1 (lipophilic antioxidant), 6.25 μM CPX (iron chelator), or 2.5 mM NAC (cysteine supplement) in H1299 (left) and FLO-1 cells (right). (**B**) Representative merged phase and red channel images of results from (A) acquired on IncuCyte. (**C**) Percentage of cell death determined by PI uptake following 24-hour treatment with 50 μM eprenetapopt in H1299 cells at indicated cell densities. (**D**) Immunoblot of Bax, Bak, Bid, Casp3, and Casp7 illustrating the efficiency of CRISPR editing in MC38 cells. (**E**) Left: Cell death in MC38 wild-type (WT) and apoptosis-deficient (Apop KO) cells following 24-hour treatment with 50 μM eprenetapopt or 12.5 μM STS. Right: Cellular metabolic activity measured by alamarBlue as a surrogate readout for cell viability following 72-hour exposure with eprenetapopt at indicated doses in wild-type and apoptosis-deficient MC38 cells. Two-way *t* test (A and E). *P < 0.05, **P < 0.01, and ****P < 0.0001. (A) *n* = 2 to 3, (D) *n* = 2, representative blots shown, and (E) *n * = 3. See also fig. S3 and movie S1.

To account for these factors, we performed ferroptosis rescue experiments and used propidium iodide (PI) uptake to directly detect dead or irreversibly damaged cells. We found that eprenetapopt-induced cell death could be reversed by ferroptosis inhibitors [ferrostatin-1 (Fer-1), lipophilic antioxidant; ciclopirox olamine (CPX), iron chelator)], but not by the poly-caspase inhibitor, zVAD-FMK, in both p53-null H1299 cells and mut-p53 expressing FLO-1 cells (Fig. 3, A and B). In contrast, zVAD-FMK blocked cell death induced by staurosporine (STS), which kills cells through the intrinsic (mitochondrial) pathway, but ferroptosis inhibitors did not (fig. S3A). These contrasting actions are consistent with ferroptosis induction by eprenetapopt. Furthermore, cells treated with eprenetapopt underwent catastrophic destabilization of their plasma membranes, without obvious visual evidence of nuclear fragmentation (movie S1). Ferroptosis inhibitors failed to restore cell proliferation of eprenetapopt-treated cells (as assessed by confluency), which likely explains our previous results using resazurin-based assays (Fig. 3, A and B) (*8*). This suggests that eprenetapopt perturbs cell proliferation beyond the induction of ferroptosis. However, *N*-acetyl-cysteine (NAC), a thiol group donor, rescued both the cell death and the proliferation defect induced by eprenetapopt (Fig. 3, A and B). This is consistent with previous studies noting that exogenously supplied thiols block eprenetapopt activity in cancer cells (*5*, *8*). We also found that increasing the cell density completely blocked the induction of cell death by eprenetapopt (Fig. 3C and movies S2, A to D), potentially due to an increased pool of GSH relative to eprenetapopt, or a non–cell autonomous intercellular contact inhibitory signal, as has been demonstrated for ferroptosis induced by cystine restriction, erastin, and GSH peroxidase 4 (GPX4) inhibitors (*23*). Wu *et al.* (*23*) demonstrated that increased cell-cell contacts with higher cell density potentiated E-cadherin–mediated activation of intracellular Merlin and Hippo signaling to suppress up-regulation of proferroptotic genes by the transcriptional coactivator, yes-associated protein (YAP).

Given that previous studies reported that eprenetapopt induces apoptosis (*5*, *24*), we tested the effect of eprenetapopt in a cell line model incapable of undergoing apoptosis to rule out the dependency on apoptotic machinery for eprenetapopt-induced cell death. To this end, we used MC38 mouse colon adenocarcinoma cells in which genes encoding the key mediators of apoptosis (Bax/Bak/Bid/Casp3/Casp7; Fig. 3D) were all disrupted. As expected, STS failed to induce significant cell death in the apoptosis-resistant cells; however, eprenetapopt was equally potent in killing both apoptosis-proficient and -resistant cells (Fig. 3E). Together, these data demonstrate that eprenetapopt induces ferroptosis and does not require apoptotic machinery to elicit cell death. This is in keeping with recent findings suggesting that eprenetapopt induces ferroptosis in AML models (*20*).

### Eprenetapopt inhibits NFS1 cysteine desulfurase activity

**Fig. 4.**
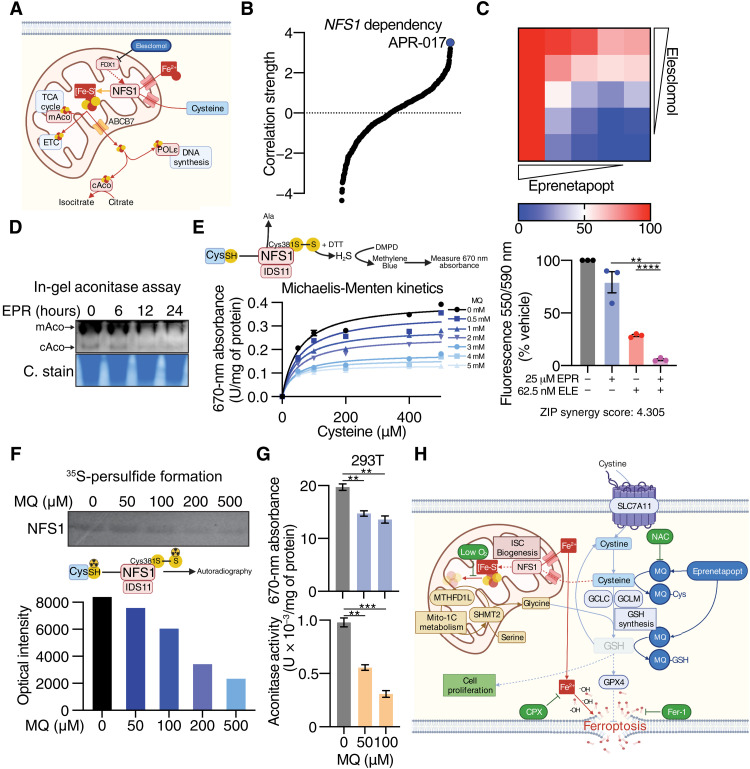
Eprenetapopt inhibits NFS1 cysteine desulfurase activity. (**A**) Schematic diagram summarizing the role of NFS1 cysteine desulfurase and important iron-sulfur cluster containing enzymes (TCA, tricarboxylic acid; ETC, electron transport chain; POLε, DNA polymerase epsilon). mAco, mitochondrial aconitase; cAco, cytosolic aconitase. (**B**) Fisher’s transformed *z*-scored Pearson correlation strength of *NFS1* gene dependency and the compound activity of the 481 CTRPv2 compounds. (**C**) Heatmap (top) and bar chart (bottom) demonstrating cell viability following 72-hour eprenetapopt and elesclomol (ELE) cotreatment in OACM5.1 cells. (**D**) In-gel aconitase assay and Coomassie stain (C. stain) (for protein loading) in OACM5.1 cells following treatment with 50 μM eprenetapopt for indicated times. (**E**) Cell-free NFS1 cysteine desulfurase assay at indicated doses of MQ and cysteine following 1-hour incubation measured by the release of hydrogen sulfide (H_2_S) by dithiothreitol (DTT) coupled to *N*,*N*-dimethyl-*p*-phenylenediamine (DMPD) hydrochloride to produce methylene blue. (**F**) Top: Two micromolars of purified NFS1 was incubated with indicated concentrations of MQ, and reaction was started with ^35^S-cysteine for 1 hour at 30°C, followed by separation on 12% nonreducing SDS–polyacrylamide gel electrophoresis (PAGE). Bottom: ImageJ quantification of the resulting bands. (**G**) Top: NFS1 cysteine desulfurase activity in human embryonic kidney 293T (293T) cells following 24-hour exposure with MQ at indicated doses measured by the release of H_2_S mediated by DTT and NFS1 cofactor pyridoxal 5′-phosphate. Bottom: Aconitase activity following 24-hour exposure with MQ at indicated doses in 293T cells. (**H**) Schematic diagram summarizing the MoA of eprenetapopt. Two-way unpaired *t* test (C and G), **P < 0.01, ***P < 0.001, and ****P < 0.0001. Error bars = SEM. (C, E, and G) *n* = 3. (D) *n* = 2, representative gel shown, and (F) *n* = 1.

Next, we sought to explain the cell proliferation defect induced by eprenetapopt. Reflecting on recent reports suggesting that depletion of CoA contributes to the induction of ferroptosis by erastin (*25*, *26*), we first referred to our metabolomics dataset (Fig. 1H); however, we found no change to CoA in eprenetapopt-treated cells (fig. S4A). Our proteomics dataset indicated a possible role of iron and mitochondrial metabolism in eprenetapopt response, including up-regulation of FDX1, a mitochondrial reductase involved in ISC biosynthesis (Fig. 1I) (*14*). We hypothesized that eprenetapopt may be inhibiting mitochondrial ISC biogenesis, likely through binding to free cysteine and limiting the cysteine desulferase activity of NFS1. NFS1 harvests sulfur from cysteine for the biogenesis of ISCs (*27*), which are important cofactors for at least 48 enzymes required to support several cell essential functions, including DNA synthesis and mitochondrial respiration (electron transport chain and tricarboxylic acid cycle) (Fig. 4A) (*28*, *29*). Furthermore, the importance of NFS1 and ISC stability in regulation of cancer cell sensitivity to ferroptosis has also been demonstrated (*30*, *31*). Supporting our hypothesis, the top compound in the CTRPv2 dataset that correlated to *NFS1* dependency was the eprenetapopt analog, APR-017 (Fig. 4B). Moreover, we observed synergy between eprenetapopt and elesclomol (Fig. 4C), where the latter has been shown to inhibit FDX1 (*32*). Eprenetapopt also reduced mitochondrial and cytosolic aconitase activity (Fig. 4D), which both rely on ISCs for their metabolic function (*33*).

Next, we used a cell-free NFS1 activity assay to demonstrate that the active agent of eprenetapopt, MQ, was able to inhibit the cysteine desulfurase activity of NFS1 in a dose-dependent fashion (Fig. 4E). Millimolar concentrations of MQ were required to inhibit NFS1 under these conditions to override the presence of 1 mM dithiothreitol (DTT) reducing agent in the reaction buffer to liberate hydrogen sulfide from the persulfide formed on NFS1 from cysteine. Therefore, we confirmed in an orthogonal fashion that MQ directly inhibits persulfide formation on NFS1 from radioactive ^35^S-cysteine (Fig. 4F). To confirm that these effects of MQ on NFS1 extend to intact cells, we also demonstrated that MQ inhibits NFS1 cysteine desulfurase activity in human embryonic kidney 293T (293T) cell lysates pretreated with MQ (Fig. 4G). This corresponded with a decrease in aconitase activity in 293T cells (Fig. 4G), without affecting the protein level of either mitochondrial aconitase or NFS1 (fig. S4B). Together, these results suggest that eprenetapopt through MQ inhibits the cysteine desulfurase activity of NFS1, likely through diminishing the availability of free cysteine by directly conjugating cysteine.

ISCs are sensitive to degradation under atmospheric oxygen conditions (standard tissue culture is 21% O_2_), and thus, cells require functional NFS1 to support ISC synthesis to maintain viability and proliferation (*30*, *34*). Conversely, ISCs are stabilized under low oxygen conditions (3% O_2_), which allows cells to proliferate in the absence of NFS1 (*30*, *34*). Knowing this, we attempted to rescue the proliferation defect induced by eprenetapopt by culturing cells (with or without Fer-1) under low oxygen conditions; however, we found that low oxygen failed to rescue the proliferation defect (fig. S4B). We found that low oxygen conditions alone did significantly decrease eprenetapopt-induced cell death, presumably through stabilization of ISCs (fig. S4B). Furthermore, under these conditions, the addition of a cyclin-dependent kinase 2 inhibitor, which was recently demonstrated to protect against the cell proliferation defect induced by suppression of the ISC-containing protein, DNA polymerase epsilon (*34*) also failed to restore proliferation (fig. S4B). Together, these results demonstrate that eprenetapopt inhibits ISC biogenesis through inhibition of NFS1 cysteine desulfurase activity, which potentiates ferroptosis induced by eprenetapopt-mediated GSH depletion. It is likely that other essential cellular functions requiring GSH as a cofactor explain the continued proliferation defect induced by eprenetapopt, even in the presence of Fer-1 and under low oxygen conditions (Fig. 4H).

## DISCUSSION

While preclinical evidence demonstrated that eprenetapopt has p53-independent anticancer activity (*20*), the disappointing preliminary results from the phase 3 clinical trial investigating eprenetapopt in combination with azacitidine highlighted the need to reexamine the patient selection strategy for eprenetapopt. Here, using a multiomics, unbiased strategy, we determine the MoA of eprenetapopt as a GSH-depleting agent that triggers ferroptosis and inhibits the cysteine desulfurase activity of NFS1. Our work supports previous findings reporting that GSH depletion and/or factors that regulate GSH availability are central determinants of eprenetapopt activity in cancer cells (*6*–*8*), as opposed to p53 and associated apoptotic machinery. This is in keeping with previous unbiased investigation of the changes to the transcriptome in response to eprenetapopt treatment in cancer cells, which detected no changes to p53 target genes but up-regulation of oxidative stress response genes, including *SLC7A11* (*13*, *35*). Further, our findings are contrary to reports concluding that eprenetapopt induces apoptosis through mut-p53 reactivation (*5*, *24*) and instead supports eprenetapopt as a ferroptosis inducer (*20*). Further, while previous reports have indicated that p53 activation may be involved in triggering ferroptosis in cancer cells (*36*), p53-null cancer cells undergo ferroptosis in response to eprenetapopt treatment, demonstrating that p53 reactivation is dispensable for ferroptosis induced by eprenetapopt. As a result, our study reframes the potential clinical utility of eprenetapopt and provides four major insights.

First, as a ferroptosis inducer, eprenetapopt remains first in class. While other clinical compounds including sorafenib (*37*), sulfasalazine (*21*), artemisinin (*38*), and statins, as well as conventional chemotherapy, immune checkpoint inhibitors, and radiotherapy (*39*), have demonstrated capacity to induce or enhance sensitivity to ferroptosis, eprenetapopt is the first compound to reach a phase 3 clinical trial where triggering ferroptosis is central to its anticancer efficacy. Furthermore, eprenetapopt may be unique, triggering an increase in turnover of GSH rather than restraining de novo GSH synthesis, as imposed by erastin through its inhibition of cystine uptake or by l-buthionine sulfoximine through inhibition of glutamate-cysteine ligase (*40*). Hematological and neurological toxicities, as well as impaired renal function, have been reported in patients receiving eprenetapopt (*3*, *4*), which is in line with reports demonstrating high sensitivity to ferroptosis among mesenchymal-derived tissues (*41*).

Second, patient selection for clinical trials and usage of eprenetapopt should not rely on *TP53* mutation. In the context of MDS, eprenetapopt is currently only indicated in ~7% of all MDS cases (*42*). However, dysregulated iron metabolism is a hallmark of a large subset of MDS with ringed sideroblasts (MDS-RS), characterized by aberrant iron accumulation in erythroblast mitochondria and accounting for ~30% of all MDS cases (*42*). The importance of this point is highlighted by the fact that mutations driving aberrant iron accumulation in MDS-RS, such as *SF3B1* mutation, rarely cooccur with *TP53* mutations (*43*). The aberrant iron accumulation in MDS-RS likely increases erythroblast sensitivity to ferroptosis. Therefore, restricting eprenetapopt to patients with *TP53*-mutated MDS, while being an area of unmet therapeutics need, may be obscuring the therapeutic benefit that eprenetapopt could provide to a wider range of patients with MDS, especially MDS-RS. As a result, reclassifying eprenetapopt as a ferroptosis inducer, therefore avoiding patient selection according to p53 status, recontextualizes the results from the phase 3 clinical trial to potentially be an example of poor patient selection rather than an indication of failure of eprenetapopt as an anticancer therapy. Both phase 1 clinical trials demonstrated that patients who responded to eprenetapopt and azacitidine had significantly improved overall survival compared to nonresponders (*3*, *4*), further highlighting the importance of patient selection.

Third, combining eprenetapopt with other agents or strategies that target cancer cell nutrient availability could provide stronger clinical efficacy. Preclinical evidence continues to grow, supporting the notion that dietary manipulation, including SG restriction, could be a powerful adjunct to conventional therapeutic approaches (*18*). To date, no attempts to test the safety or efficacy of SG restriction in patients with cancer have been undertaken. Here, we show that priming tumors with SG restriction improves the efficacy of eprenetapopt in esophageal cancer models. A very recent study also demonstrated synergy between eprenetapopt and asparaginase (an enzyme that degrades asparagine) in acute lymphoblastic leukemia cell lines (*44*), which could hint that GSH depletion by eprenetapopt more generally increases cancer cell dependency on exogenous amino acid uptake. Furthermore, the combination of venetoclax (inhibitor of the anti-apoptopic protein BCL-2) and azacitidine has been shown to target mitochondrial metabolism through inhibition of the glutathionylation of complex II of the electron transport chain (*45*) and diminishes the uptake of global amino acids in primary patient AML stem cells (*46*). Moreover, depleting GSH through exogenous cyst(e)ine degradation with cyst(e)inase demonstrated a similar MoA in AML stem cells (*47*). Together with our study, these studies provide a strong rationale for the combination of eprenetapopt, venetoclax, and azacitidine, which is currently under clinical investigation in a phase 1/2 study in myeloid malignancies (*48*). Preliminary results from this phase 1/2 clinical trial report that the study successfully reached its primary end point with a CR rate of 37% and found that the triple combination of eprenetapopt, venetoclax, and azacitidine was well tolerated in patients (www.aprea.com). Further study should be undertaken to determine whether the response to the triple combination of eprenetapopt, venetoclax, and azacitidine could be improved with SG dietary restriction.

Fourth, the limited efficacy demonstrated by eprenetapopt monotherapy in both of our esophageal cancer models illuminates important considerations for clinical utility of eprenetapopt in the solid tumor setting. Previous studies have demonstrated the limitations of targeting GSH synthesis pathways either genetically via GCLM suppression or pharmacologically with buthionine sulfoximine in solid tumor models (*49*). Furthermore, we also found that eprenetapopt efficacy in cell culture can be limited by high cell confluency and low O_2_ levels—two environmental factors that are commonplace in solid malignancies. Given that both erastin and cystine restriction exhibit similar phenotypes in solid tumors (*23*), identifying novel ferroptosis inducers or modulators that overcome these factors will be critical for developing ferroptosis inducers suitable for use in patients with advanced solid tumors.

In summary, our study demonstrates that eprenetapopt targets cancer cells through GSH depletion and inhibiting cysteine desulfurase activity of NFS1, leading to iron-dependent, nonapoptotic ferroptosis. The precise mechanism by which eprenetapopt depletes GSH and inhibits NFS1 remains to be determined and thus is a limitation of the study. Nevertheless, this work not only details novel determinants of eprenetapopt activity in cancer cells, including mito-1C metabolism enzymes, but also provides a clinical roadmap for targeting antioxidant pathways in tumors with eprenetapopt, beyond *TP53*-mutant tumors.

## MATERIALS AND METHODS

### Compounds and reagents

Eprenetapopt was provided by Aprea Therapeutics Inc. (Boston, Massachusetts, USA). Elesclomol, erastin, STS, and SNS-032 were from Selleckchem (Houston, Texas, USA). MQ, sodium formate, glycine, serine, NAC, Fer-1, zVAD-FMK, and CPX were from Sigma-Aldrich (St. Louis, Missouri, USA).

### Cell cultures

NCI-H1299 (H1299) (RRID:CVCL_0060), OACM5.1 (RRID:CVCL_1842), FLO-1 (RRID:CVCL_2045), and 293T (RRID:CVCL_0063) cells were from American Type Culture Collection. All cells were maintained at 37°C with 5% CO_2_, and their identities were authenticated by short tandem repeat analysis using a PowerPlex 16 genotyping system (Promega) and confirmed mycoplasma free by polymerase chain reaction (PCR) every 2 months (Cerberus Sciences). Cells were thawed from early passage stocks and passaged for no more than 4 months. Unless otherwise specified, all culture media contained 10% fetal bovine serum (FBS) supplemented with penicillin (50 U/ml) and streptomycin (50 mg/ml) (Life Technologies). MC38, FLO-1, and HEK-239T cells were grown in Dulbecco’s modified Eagle’s medium (DMEM) containing 2.5 mM l-glutamine and d-glucose (Life Technologies). MC38 cells were maintained in 10% CO_2_. H1299 and OACM5.1 cells were grown in RPMI 1640 containing 2.5 mM l-glutamine.

### CRISPR screening

OACM5.1 cells were engineered to express the Cas9 endonuclease by transduction with the FUCas9Cherry vector (Addgene, #70182) and subsequent selection for mCherry-positive cells, or Cas9 endonuclease dead (dCas9) by transduction with the dCas9-VP64_Blast vector (Addgene, #61425) and subsequent selection for blasticidin resistance (10 μg/ml). For genome-wide CRISPRko and CRISPRa screening, OACM5.1 cells expressing Cas9 or dCas9-VP64 were transduced with a genome-wide sgRNA Brunello [76,441 sgRNAs, Addgene, #73178 (*10*)] or Calabrese [56,762 sgRNAs, Addgene, #92379 (*11*)] library, respectively, at a multiplicity of infection of 0.3, aiming for 500-fold starting representation of each guide. Puromycin selection (1 μg/ml) was applied for 7 days, and surviving cells were subsequently split into relevant treatment conditions (CRISPRko, vehicle versus 10 μM eprenetapopt; CRISPRa, vehicle versus 15 μM eprenetapopt; see fig. S1 for effect of eprenetapopt on cell number). Cells were passaged every 4 days in T175 flasks with 2 × 10^6^ cells per flask for a total of 8 days for the CRISPRko screen and 28 days for the CRISPRa screen. Genomic DNA was extracted using the DNeasy Blood and Tissue Kit (QIAGEN), and sequencing libraries were generated by PCR as previously detailed (*50*). The libraries were sequenced on a NextSeq 500 (Illumina) to generate 75–base pair single-end reads, which were demultiplexed with Bcl2fastq (v2.17.1.14). The sequencing reads were subsequently trimmed with Cutadapt (v1.7) to remove sgRNA vector–derived sequences, then sgRNA reads were counted, and the distribution was analyzed using MAGeCK (v0.5.8) using read depth (--norm-method total) normalization. MAGeCK score was determined by −log_10_(neg *P* value) + log_10_(pos *P* value), and datasets were visualized on GraphPad Prism (v9.0). Validation experiments were performed on polyclonal OACM5.1 cells expressing individual sgRNAs (table S1) following selection with puromycin. Formate and glycine rescue experiments were performed under conditions matching the CRISPR screening procedure scaled down to T25 flask. SG auxotrophy experiments were performed in 96-well plates using SG-free medium supplemented with dialyzed FBS.

### DepMap analysis

Cancer cell line gene dependency data (DepMap Public 21Q1 release) and APR-017 cancer cell line sensitivity data (CTRPv2) were obtained from the DepMap web portal (www.depmap.org), and correlation analyses were performed in RStudio (v1.1.4) using the *cor.test* function. Correlation strengths were determined by adjusting the Pearson correlations with a Fisher’s *z*-transformation to account for the differences in cell lines analyzed.

### Metabolomics profiling

OACM5.1 cells (3.3 × 10^5^) were plated in six-well plates, allowed to adhere for 24 hours, and subsequently treated with vehicle or 50 μM eprenetapopt for 12 hours in fresh medium (six replicates per condition). Medium was aspirated, and cells were washed with water (at 37°C) for 10 s and then snap-frozen by the addition of liquid nitrogen directly to tissue culture plates. Metabolite extraction was performed with ice-cold methanol/chloroform (9:1, v/v) solvent containing ^13^C-sorbitol, ^13^C,^15^N-valine, ^13^C,^15^N–adenosine 5′-monophosphate, and ^13^C,^15^N–uridine 5′-monophosphate as internal standards. Metabolite-containing supernatants were collected following centrifugation at 16,000*g* for 5 min to pellet insoluble material and debris.

Metabolite analysis was performed using an Agilent 6520 series quadrupole time-of-flight mass spectrometer (QTOF MS) (Agilent Technologies) with chromatographic separation on an Agilent 1200 series high-performance LC (HPLC) system (Agilent Technologies). Metabolite separation was performed on a Merck SeQuant ZIC-HILIC column (150 mm by 2.1 mm, 5-μm particle size) using solvent A, 20 mM ammonium carbonate (pH 9.0; Sigma-Aldrich), and solvent B, 100% acetonitrile. The gradients used were as follows: negative mode—time = 0 min, 90% B; *t* = 0.5 min, 90% B; *t* = 12 min, 40% B; *t* = 14 min, 40% B; *t* = 15 min, 5%; *t* = 18 min, 5% B; and *t* = 19 min, 90%. Samples were stored in the autosampler at 4°C and 7 μl injected onto the column, which was maintained at 40°C, with a solvent flow rate of 250 μl/min.

MS analysis was performed on an Agilent 6545 series QTOF MS (Agilent Technologies) as described previously (*51*). Negative mode LC-MS data were collected in centroid mode with a scan range of 50 to 1700 mass-to-charge ratio (*m/z*) and an acquisition rate of 1.2 spectra/s. Samples were analyzed in the same analytical batch and randomized with a quality control every five samples. Thirteen mixtures of authentic standards (550 metabolites) were also run to generate the library for targeted analysis. Level 1 metabolite identification [according to the Metabolite Standard Initiative (*52*)] was based on matching accurate mass, retention time, and tandem MS (MS/MS) spectra to the 550 authentic standards in the MA in-house library. Metabolite abundance based on area under the curve (AUC) was obtained using Agilent Masshunter Quantitative Analysis B 0.7.00. Statistical analysis was performed applying the web-based platform MetaboAnalyst applying no missing value imputation, normalization to median peak area, and no scaling or transformation (*53*).

For isotopic tracing analysis, OACM5.1 cells were prepared in the same fashion as steady-state experiments; however, cells were cultured in cystine-free RPMI 1640 (Sigma-Aldrich) with 10% dialyzed FBS supplemented with 208 μM l-[3′,3′-^13^C_2_]-cystine or l-^12^C-cystine (three replicates per condition). For enhanced resolution, identification and detection of the isotopic species metabolite analysis were performed on the Vanquish Horizon UHPLC System, integrated biocompatible system coupled to Thermo Fisher Scientific Orbitrap ID-X Tribrid mass spectrometer (Thermo Fisher Scientific, San Jose). The polar metabolite chromatographic separation was performed on the Metabolomics Australia Merck SeQuant ZIC-HILIC column (150 mm by 4.6 mm, 5-μm particle size) method previously with modifications.

Briefly, the mobile phase of A [20 mM ammonium carbonate (pH 9.0; Sigma-Aldrich)] and B (100% acetonitrile) was run at a flow rate of 300 μl/min with the following gradient: time = 0.0 min, 80% B; *t* = 0.5 min, 80% B; *t* = 15.5 min, 50% B; *t* = 17.5 min, 30% B; *t* = 18.5 min, 5%; *t* = 21.0 min, 5% B; and *t* = 28 min, 80%. Samples were stored in the autosampler at 4°C and 7 μl injected onto the column maintained at 30°C. Full-scan (MSn = 1) Orbitrap ID-X data were acquired in positive and negative ionization modes separately within the mass range of 70 to 1300 *m/z* at 240,000 resolution. Ion source parameters were set as follows: sheath gas = 40 arbitrary units, Aux gas = 10 arbitrary units, sweep gas = 1 arbitrary unit, spray voltage = 3.5 kV (positive ion)/3.2 kV (negative ion), capillary temperature = 300°C, and vaporizer temperature = 400°C. Metabolite and isotope abundance are based on AUC deconvoluted from the labeling data using the “detect and analyze labeled compounds” module and in-house mass list matching in Compound Discoverer 3.2. A mass error of 3 parts per million and a retention time range of 0.3 s in feature grouping and molecular formula and metabolite matching were applied. All molecules were annotated according to guidelines for reporting of chemical analysis results as proposed in Metabolomics Standards Initiative level 1 (*52*).

### Quantitative proteomics

OACM5.1 cells (5.0 × 10^6^) were plated in 15-cm plates, allowed to adhere for 24 hours, and subsequently treated with vehicle or 50 μM eprenetapopt for 12 hours in fresh medium (four replicates per condition). Medium was aspirated, and then cells were washed with ice-cold phosphate-buffered saline (PBS) and lysed at 4°C in radioimmunoprecipitation assay (RIPA) buffer (1 mM EDTA, 1% NP-40, 0.5% sodium deoxycholate, 0.1% SDS, 50 mM sodium fluoride, and 1 mM sodium pyrophosphate in PBS) mixed with protease and phosphatase inhibitors (Roche). Debris was pelleted by centrifugation at 11,000*g* for 10 min. Overnight acetone precipitation of proteins was performed at −20°C to purify the samples. Proteins were resuspended in 8 M urea in 50 mM triethylammonium bicarbonate (pH 8), reduced with 10 mM tris(2-carboxyethy)phosphine (Sigma-Aldrich), alkylated with 55 mM iodoacetamide (Sigma-Aldrich), and subjected to tryptic digest with Pierce Trypsin Protease MS-Grade (Thermo Fisher Scientific) overnight at 37°C (1:50 trypsin to protein). Formic acid (1%, v/v) was added to acidify the solution before loading onto Oasis cartridges (Waters). Peptides were eluted in 80% (v/v) acetonitrile and 0.1% (v/v) trifluoroacetic acid, freeze-dried, and stored at −80°C until analyzed by LC-MS/MS.

LC-MS/MS was carried out on a QExactive plus Orbitrap mass spectrometer (Thermo Fisher Scientific) with a nanoESI interface in conjunction with an Ultimate 3000 RSLC nanoHPLC (Dionex Ultimate 3000). The LC system was equipped with an Acclaim Pepmap nano-trap column (Dinoex-C18, 100 Å, 75 μm by 2 cm) and an Acclaim Pepmap RSLC analytical column (Dinoex-C18, 100 Å, 75 μm by 50 cm). The tryptic peptides were injected to the enrichment column at an isocratic flow of 5 μl/min of 2% (v/v) CH_3_CN containing 0.1% (v/v) formic acid for 5 min applied before the enrichment column was switched in-line with the analytical column. The eluents were 5% dimethyl sulfoxide (DMSO) in 0.1% (v/v) formic acid (solvent A) and 5% DMSO in 100% (v/v) CH_3_CN and 0.1% (v/v) formic acid (solvent B). The flow gradient was (i) 0 to 6 min at 3% solvent B, (ii) 6 to 95 min at 3 to 22% solvent B, (iii) 95 to 105 min at 22 to 40% solvent B, (iv) 105 to 110 min at 40 to 80% solvent B, (v) 110 to 115 min at 80 to 80% solvent B, and (vi) 115 to 117 min at 80 to 3% and was equilibrated at 3% solvent B for 10 min before the next sample injection. The QExactive plus mass spectrometer was operated in the data-dependent mode, whereby full MS1 spectra were acquired in positive mode, 70,000 resolution, an automatic target control target of 3 × 10^6^, and a maximum injection time of 50 ms. Fifteen of the most intense peptide ions with charge states of ≥2 and an intensity threshold of 1.7 × 10^4^ were isolated for MS/MS. The isolation window was set at 1.2 *m/z*, and precursors were fragmented using normalized collision energy of 30, 17, and 500 resolution, an automatic target control target of 1 × 10^5^, and a maximum injection time of 100 ms. Dynamic exclusion was set to be 30 s.

### Xenograft models and treatments

All animal experiments were approved by the Peter MacCallum Cancer Centre Animal Experimentation Ethics Committee and undertaken in accordance with the National Health and Medical Research Council Australian Code of Practise for the Care and Use of Animals for Scientific Purposes. For FLO-1 LM cell line xenografts (*19*), 5 × 10^6^ cells suspended in 100 μl of 1:1 PBS and Matrigel (BD Biosciences) were subcutaneously injected into the right flank of ~6 week-old female nonobese diabetic–severe combined immunodeficient interleukin-2Rγ^KO^ (NSG) mice. PDXs were established and implanted into a dorsal intramuscular pocket of NSG mice as previously described (*54*). Mice were randomized to SG deplete or control chow ad libitum (AIN93G rodent diet, Specialty Feeds, Australia) and dosed with eprenetapopt (100 mg/kg) or 0.9% saline, intraperitoneally injected daily, once tumors reached 100 mm^3^. Tumor volume was assessed blinded to treatment group with caliper measurements every 3 to 4 days and calculated using the formula (length × weight^2^)/2. Metastatic spread was determined by bioluminescence imaging as previously described (*19*) involving weekly monitoring using the Xenogen IVIS 100 Imaging System (Caliper Life Science). At experimental end point (tumor volume > 1400 mm^3^), the whole mouse and its organs were imaged to determine the extent and distribution of metastases. Tumors were weighed and tumor growth inhibition was calculated with the formula [1 − (Tf − Ti)/mean(Cf − Ci)] × 100, where Tf, Ti, Cf, and Ci represent final (f) and initial (i) tumor volume of drug treated (T) and control (C) animals, respectively.

### Cell death

For ferroptosis rescue experiments, cells were cotreated with rescue compounds (Fer-1, CPX, zVAD-FMK, and NAC) in medium containing PI in 96-well plates (FLO-1, 5000 cells per well; H1299, 3000 cells per well). Cell death and confluency were quantified using an IncuCyte FLR (Essen BioScience) imaging phase and red channels. Percentage (%) of cell death was determined by dividing the percentage of red confluency by the percentage of phase confluency. Note that 100% cell death was ascribed if percentage of red confluency was greater than phase confluency.

### Cell viability

For dose-response assays, 10-point log_2_ serial dilutions of eprenetapopt were added to 96-well plates containing cells (OACM5.1, 10,000 cells per well; Mc38, 10,000 cells per well). After 72-hour incubation, cell viability was determined using alamarBlue reagent (Life Technologies), and fluorescence was read at 550 nm/590 nm on a Cytation 3 Imaging Reader (BioTek).

### Expression and purification of human NFS1 and ISD11

*E. coli* BL21(DE3) cells were cotransformed with pET15b-NFS1∆55-His_6_ encoding a truncated human NFS1 N-terminal 6×His-tagged fusion protein without the first 55 amino acids and pET15b-ISD11-His_6_ encoding full-length human 6×His-tagged ISD11 N-terminal fusion protein (Novagen) (*55*). Transformed cell cultures were grown at 30°C for 1 liter of LB medium containing ampicillin (150 μg/ml) and chloramphenicol (50 μg/ml). NFS1/ISD11 coexpression was induced by the addition of 200 μM isopropyl-β-d-thiogalactoside, followed by continued growth for 16 hours at 16°C. Cells were harvested by centrifugation at 8000*g*. After cell lysis, the soluble fraction was transferred onto a column with Nickel–nitrilotriacetic acid (QIAGEN). NFS1/ISD11 was coeluted with 50 mM NaH_2_PO_4_, 300 mM NaCl, and 250 mM imidazole buffer (pH 8.0) containing 50 μM pyridoxal phosphate (PLP) and 10% (v/v) glycerol. Final purification of NFS1/ISD11 was achieved using size exclusion chromatography with a Superdex 200 column (GE Healthcare) equilibrated with 50 mM tris, 200 mM NaCl, and 10 μM PLP (pH 8.0).

### In vitro NFS1 cysteine desulfurase activity

Cysteine desulfurase activity of NFS1/ISD11 was quantified the by the methylene blue method (*56*) as previously described with some amendments (*55*). Assay mixtures in a total volume of 0.8 ml containing 50 mM tris, 200 mM NaCl, 10 μM PLP, 1 mM DTT (pH 8.0), and 2 μM NFS1/ISD11. Varying concentrations of the active agent of eprenetapopt, MQ (0.5 to 5 mM), were added to the mixture and followed by varying concentrations of cysteine (50 to 500 μM) to initiate the reactions. Mixtures were incubated for 1 hour at 37°C before the reactions were quenched by the addition of 100 μl 20 mM *N*,*N*-dimethyl-*p*-phenylenediamine (DMPD) in 7.2 M HCl and 100 μl of 30 mM FeCl_3_ in 1.2 M HCl. The resulting solution was incubated for a further 20 min for color development and subsequently spun at 13,000*g* for 5 min. The supernatants were transferred to cuvettes, and methylene blue content was determined at 670 nm using a Varioskan Flash (Thermo Fisher Scientific).

### In-cell NFS1 cysteine desulfurase activity

293T cells were plated in T75 flasks and treated with MQ (0, 50, and 100 μM) for 24 hours. Cells were detached with ice-cold trypsin, washed twice with ice-cold PBS before being resuspended in lysis buffer [0.1% NP-40, 50 mM tris-EDTA (pH 8.0)] for 30 min at 4°C, and spun at 13,000*g* for 15 min. The protein concentration of the supernatants was quantified, and 2.5 mg of each protein lysate was combined with 1 mM DTT, 10 μM PLP, and 500 μM cysteine in a final volume of 400 μl for 1 hour at 37°C. As above, DMPD and FeCl_3_ were added to stop the reaction, and the generated methylene blue content was determined at 670 nm.

### Inhibition of persulfide formation on l-cysteine desulfurase

Purified NFS1/ISD11 was radiolabeled with ^35^S-cysteine (Cambridge Isotopes) for persulfide formation as adapted from (*57*). Briefly, 2 μM NFS1/ISD11 was incubated with varying concentrations of MQ (50 to 500 μM) in reaction buffer containing 50 mM tris, 200 mM NaCl, and 150 mM KCl in a total volume of 35 μl. The reaction was started with 10 μCi ^35^S-cysteine (1075 Ci/mmol) and incubated 30°C for 1 hour. Resulting solutions were mixed with SDS loading buffer without boiling and separated on a 12% SDS–polyacrylamide gel electrophoresis (PAGE) under nonreducing conditions. Proteins were analyzed by autoradiography.

### In-gel aconitase assay and aconitase activity assay

OACM5.1 cells (5.0 × 10^6^) were plated in 15-cm plates, allowed to adhere for 24 hours, and subsequently treated with 50 μM eprenetapopt for 0, 6, 12 and 24 hours in fresh medium. Cells were collected and resuspended in lysis buffer containing 1% Triton X-100, and protein content was determined. As previously described (*33*), aconitase activity gels were composed of a separating gel containing 8% acrylamide, 132 mM tris base, 132 mM borate, 3.6 mM citrate, and a stacking gel containing 4% acrylamide, 67 mM tris base, 67 mM borate, and 3.6 mM citrate. The running buffer contained 25 mM tris (pH 8.3), 192 mM glycine, and 3.6 mM citrate. Samples contain cell lysates of equal protein content (~100 μg), 25 mM tris-Cl (pH 8.0), 10% glycerol, and 0.025% bromophenol blue. Electrophoresis was carried out at 170 V at 4°C. Aconitase activities were assayed by incubating the gel in the dark at 37°C for 45 min in 100 mM tris (pH 8.0), 1 mM nicotinamide adenine dinucleotide phosphate (NADP^+^), 2.5 mM *cis*-aconitic acid, 5 mM MgCl_2_, 1.2 mM thiazolyl blue tetrazolium bromide (MTT), 0.3 mM phenazine methosulfate, and isocitrate dehydrogenase (5 U/ml) (all from Sigma-Aldrich). Gels were imaged on a Bio-Rad ChemiDoc, then stained with Coomassie to verify equal protein loading, and imaged again.

For 293T cells, 50 μl of cell lysates prepared for the in-cell NFS1 activity assay were incubated with 250 μl of R1 buffer [50 mM tris-HCl, 50 mM NaCl, 5 mM MgCl_2_, 0.5 mM NADP^+^, and 0.01 U of isocitrate dehydrogenase (pH 8)] for 5 min at 37°C. A total of 200 μl of R2 buffer [50 mM tris-HCl, 50 mM NaCl, 5 mM MgCl_2_, and 2.5 mM *cis*-aconitic acid (pH 8)] was added to start the reaction which was monitored for 3 min at 340 nm using Shimadzu UV-Vis Spectrophotometer UV-2600.

### Immunoblot

As per established protocols (*8*, *58*), cells were lysed at 4°C in RIPA buffer (1 mM EDTA, 1% NP-40, 0.5% sodium deoxycholate, 0.1% SDS, 50 mM sodium fluoride, and 1 mM sodium pyrophosphate in PBS) mixed with protease and phosphatase inhibitors (Roche). Equal amounts of protein were boiled, resolved by SDS-PAGE, and transferred to polyvinylidene difluoride membranes. For total protein glutathionylation detection, lysates were not boiled and were resolved under nonreducing conditions. Membranes were incubated in blocking buffer [5% skim milk in 0.05% tris-buffered saline with 0.05% Tween 20 (TBS-T)] for 1 hour at room temperature and probed overnight in primary antibody at 4°C. Blots were washed thrice in 0.05% TBS-T, followed by incubation with peroxidase-conjugated secondary antibody (Dako) for 1 hour at room temperature. Protein levels were detected using Amersham ECL Western Blotting Detection reagents (GE Healthcare) or ECL Plus Western blotting substrate kit (Thermo Fisher Scientific). Antibodies are detailed in table S2.

### Generating apoptosis-resistant MC38 cells

Gene disruption was carried out using CRISPR-Cas9 technology. Each synthetic gRNA (two gRNAs per gene; 150 pmol) specifically targeting BAX, BAK1, BID, CASP3, or CASP7 (Synthego; table S3) in a volume of 0.5 μl were pooled in a final volume of 5 μl. A total of 200 pmol of Alt-R S.p. HiFi Cas9 Nuclease in 2-μl volume (Integrated DNA Technologies) was then added, mixed, and incubated at room temperature for 10 min to allow formation of Cas9/gRNA complexes. Meanwhile, 3 × 10^5^ MC38 cells for each experimental condition (harvested at 40% confluency, subcultured 2 days prior) were washed ×3 in room-temperature PBS, followed by resuspension in 20 μl of Amaxa Buffer SF. Resuspended cells were then mixed with Cas9/gRNA complexes, transferred to a 16-well electroporation stripette (Amaxa 4D, Lonza), and electroporated using program CM-130. Immediately after electroporation, 150 μl of prewarmed medium (DMEM, 10% FBS, and penicillin-streptomycin) was added to each well and allowed to recover in an incubator for 15 min. Cells were then transferred to six-well plates. After at least 5 days, the effect of gene disruption on protein levels was evaluated by Western blot.

### Radioactive cystine uptake assay

*SLC7A11* was ectopically expressed in H1299 using the GE Dharmacon Precision LentiORF pLOC downstream of the cytomegalovirus promoter and contains turbo–green fluorescent protein as a reporter gene. H1299 SLC7A11-overexpressing cells were washed in prewarmed cystine-free RPMI 1640 (uptake buffer). At this point, in each well, the cystine-free-medium was replaced with 300-μl uptake buffer containing eprenetapopt (preheated at 90°C for 30 min to hydrolyze to MQ) or erastin with 0.04 μCi of l-[1,2,1′,2′-^14^C]-cystine (PerkinElmer) for 10 min at room temperature. Cells were then washed thrice with ice-cold cystine-free medium and lysed in 400 μl of 0.2 M NaOH with 1% SDS. This lysate was added to 4 ml of scintillation fluid, and radioactive counts per minute were obtained using a scintillation counter.

### Gene expression with quantitative reverse transcription PCR

Total RNA was extracted using NucleoSpin RNA kit (Macherey-Negal), cDNA was synthesized from 1 μg of RNA using the High-capacity cDNA Reverse Transcription Kit (Applied Biosystems), and gene expression was determined by SYBR-green reverse transcription PCR on a LightCycler 480 (Roche). Target gene expression was normalized to *ACTB* and analyzed using the ΔΔ*C*_t_ method. Primer sequences are detailed in table S4.

### Statistical analysis and replication

Data are presented as means ± SEM and analyzed by Student’s *t* test unless otherwise indicated. GI_50_ dose (where 50% growth inhibition is achieved) of single agents was determined by fitting the Hill equation. Statistical analyses and data presentation were performed using Prism 9 (GraphPad). No power analyses were performed to determine sample size before experimentation. Blinding was undertaken during in vivo experiments.

## Notes

View/request a protocol for this paper from *Bio-protocol*.
