# Supplementary material for: Eprenetapopt triggers ferroptosis, inhibits NFS1 cysteine desulfurase, and synergizes with serine and glycine dietary restriction
Source: Sci Adv. 2022 Sep 14;8(37):eabm9427. doi: 10.1126/sciadv.abm9427 (PMC9473576; doi:10.1126/sciadv.abm9427)
Supplement: Supplementary file 1 — Figs. S1 to S4 Tables S1 to S4 [file sciadv.abm9427_sm.pdf]

Science Advances
AAAS

Supplementary Materials for

Eprenetapopt triggers ferroptosis, inhibits NFS1 cysteine desulfurase, and synergizes with serine and glycine dietary restriction

Kenji M. Fujihara *et al.*

Corresponding author: Nicholas J. Clemons, nicholas.clemons@petermac.org;
Kenji M. Fujihara, kenji.fujihara@petermac.org

Sci. Adv. **8**, eabm9427 (2022)
DOI: 10.1126/sciadv.abm9427

The PDF file includes:

Figs. S1 to S4
Tables S1 to S4
Legend for dataset S1
Legends for movies S1 and S2

Other Supplementary Material for this manuscript includes the following:

Dataset S1
Movies S1 and S2

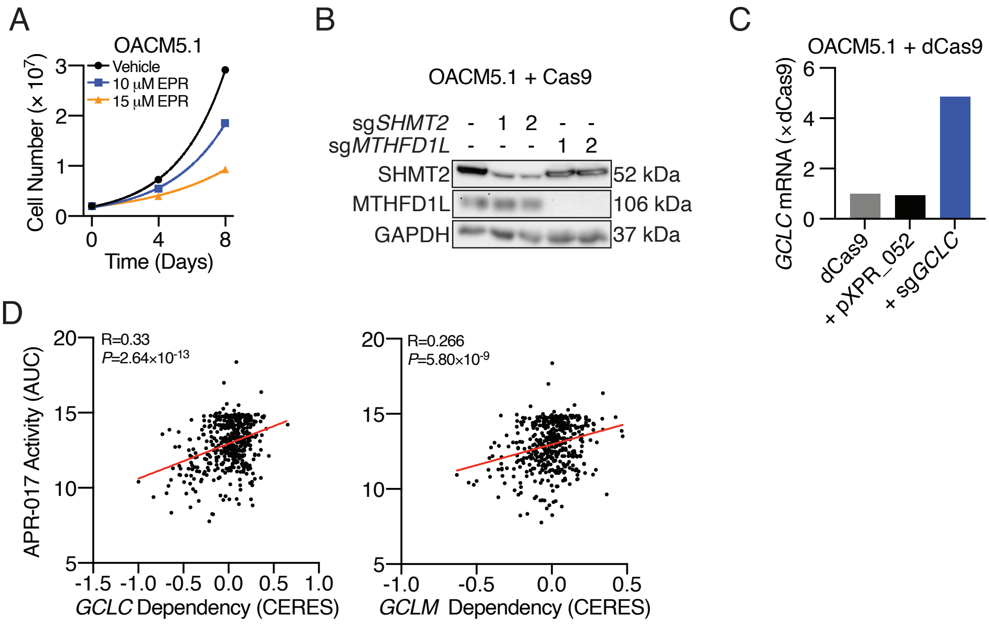

Figure S1. Related to Figure 1 Multiomics strategy to determine the mechanism of action of eprenetapopt

(A) Representative growth curve of OACM5.1 cells treated with eprenetapopt at indicated doses over 8 days. **(B)** Immunoblot of SHMT2 and MTHFD1L illustrating the efficiency of two independent sgRNA guides in bulk OACM5.1 cells following puromycin selection. **(C)** Relative gene expression of *GCLC* in OACM5.1 dCas9 cells expressing CRISPR activation sgRNA guide for *GCLC* or control guide. **(D)** Scatterplots correlating eprenetapopt analogue (APR-017) activity from CTRPv2 with CCL dependency on *GCLC* and *GCLM* in DepMap. (CERES, copy-number adjusted gene dependency score). Pearson's correlation **(D)**. **(A,B,C)** N=1. **(D)** N=466.

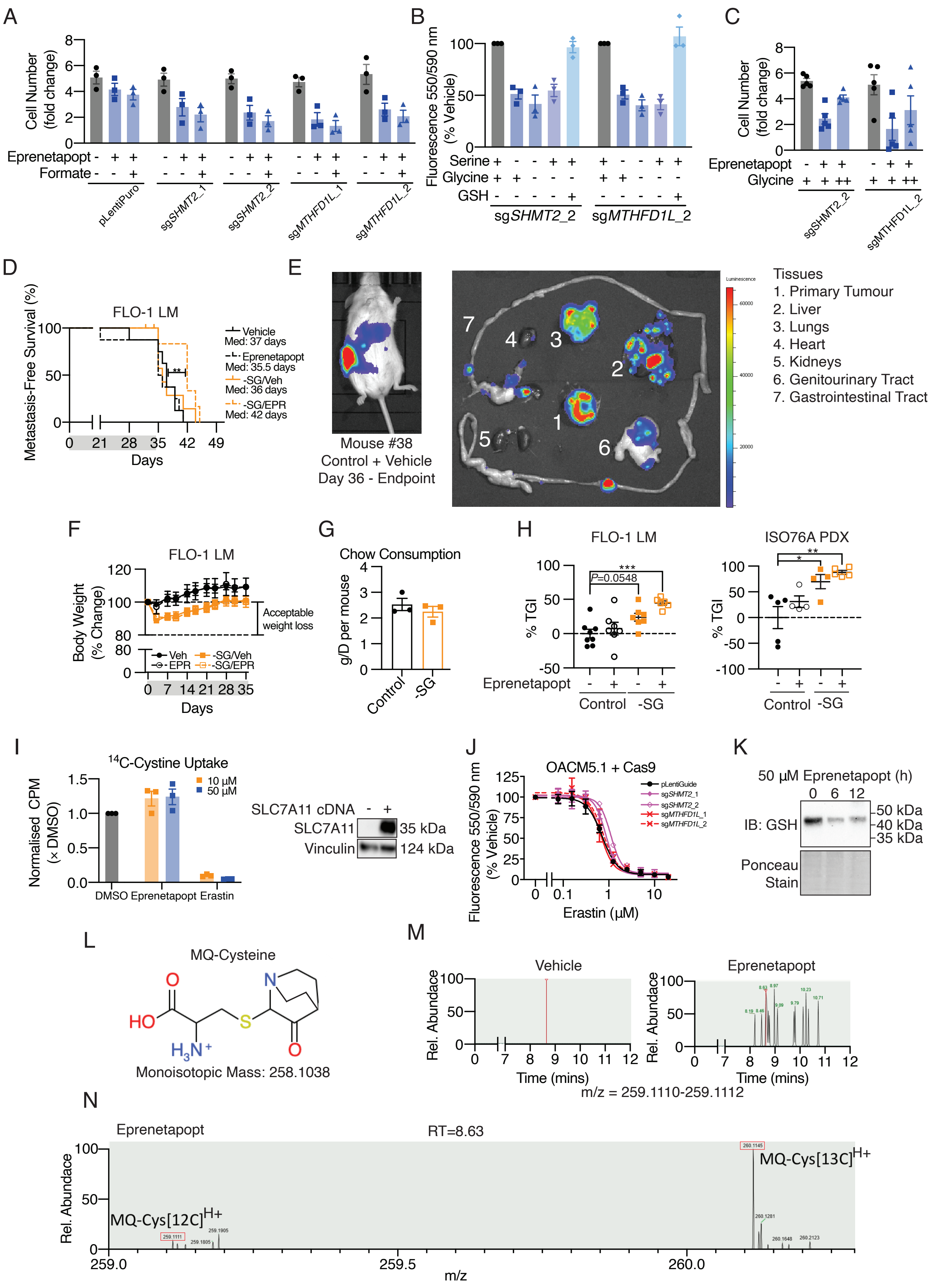
A
B
C
D
E
F
G
H
I
J
K
L
M
N
Cell Number (fold change)
Eprenetapopt
Formate
pLentiPuro
sgSHMT2_1
sgSHMT2_2
sgMTHFD1L_1
sgMTHFD1L_2
Fluorescence 550/590 nm (% Vehicle)
Serine
Glycine
GSH
FLO-1 LM
Metastasis-Free Survival (%)
Vehicle Med: 37 days
Eprenetapopt Med: 35.5 days
-SG/Veh Med: 36 days
-SG/EPR Med: 42 days
Days
Mouse #38
Control + Vehicle
Day 36 - Endpoint
Tissues
1. Primary Tumour
2. Liver
3. Lungs
4. Heart
5. Kidneys
6. Genitourinary Tract
7. Gastrointestinal Tract
Body Weight (% Change)
Acceptable weight loss
Chow Consumption
g/D per mouse
Control
-SG
ISO76A PDX
% TGI
P=0.0548
14C-Cystine Uptake
Normalised CPM (× DMSO)
10 µM
50 µM
DMSO Eprenetapopt Erastin
SLC7A11 cDNA
SLC7A11 35 kDa
Vinculin 124 kDa
OACM5.1 + Cas9
pLentiGuide
Erastin (µM)
50 µM Eprenetapopt (h)
IB: GSH
Ponceau Stain
50 kDa
40 kDa
35 kDa
MQ-Cysteine
Monoisotopic Mass: 258.1038
Vehicle
Rel. Abundace
Time (mins)
m/z = 259.1110-259.1112
RT=8.63
MQ-Cys[12C]H+
MQ-Cys[13C]H+
m/z

Figure S2. Relating to Figure 2 Eprenetapopt and mitochondrial one-carbon metabolism

(A) Relative cell number following treatment with 10 μM eprenetapopt ± 1 mM formate supplementation for 4 days in OACM5.1 cells transduced with control (pLentiPuro) or *SHMT2* or *MTHFD1L* sgRNA. **(B)** Cell viability compared to complete media (CM) following 72 h of serine, glycine or serine and glycine deprivation, and glycine deprivation rescued with 1 mM GSH-monoethyl ester (GSH) in OACM5.1 cells transduced with *SHMT2 or MTHDF1L* sgRNA_2. **(C)** Relative cell number following treatment with 10 μM eprenetapopt ± 1 mM glycine supplementation for 4 days in OACM5.1 cells transduced with *SHMT2 or MTHDF1L* sgRNA_2. **(D)** Metastasis-free survival of NSG mice inoculated with FLO-1 LM tumours treated with eprenetapopt (EPR, 100 mg/kg, daily) or vehicle on either normal or SG-free diets for 35 days. **(E)** Representative bioluminescence images of the distribution of metastatic lesions before and after dissection of a NSG mouse at ethical endpoint. **(F)** % change to body weight of mice over treatment period. Loss of ≤20% body weight relative to beginning of treatment period was considered ethically acceptable. **(G)** Average daily chow consumption of mice on the indicated diets over 7 days. **(H)** % tumour growth inhibition quantified at the end of treatment (35 days for FLO-1 LM, 21 days for PDX). **(I)** Left: Relative radioactive cystine uptake in H1299-SLC7A11 overexpressing cells co-treated with DMSO (as control), pre-heated eprenetapopt and erastin (known cystine uptake inhibitor) at indicated doses. (CPM, counts per minute) Right: Immunoblot demonstrating overexpression of SLC7A11 in H1299 used for cystine uptake assay. **(J)** Cell viability following 72 h exposure with erastin at indicated doses in OACM5.1 cells transduced with indicated sgRNAs. **(K)** Changes to total glutathionylation level following 50 μM eprenetapopt treatment at indicated time points in H1299 cells. **(L)** Chemical structure and mass of MQ-conjugated cysteine. **(M)** Scan to identify MQ-cysteine from LC-MS run of OACM5.1 treated with ^{13}C-cystine and 50 μM eprenetapopt or vehicle for 12 h, over 12 mins of retention time. **(N)** Scan indicating relative abundance of species from retention time (RT) = 8.63, from LC-MS run of OACM5.1 treated with ^{13}C-cystine and 50 μM eprenetapopt. MQ-Cys[^{12}C]$^{H+}$ denotes MQ-cysteine from endogenous ^{12}C-cysteine and MQ-Cys[^{13}C]$^{H+}$ denotes MQ-cysteine from exogenous ^{13}C-cysteine. Log-rank (Mantel-Cox) test **(D)**, one-way ANOVA with Dunnett's multiple comparisons test **(H)**. Error bars = SEM. **(A,B,E,I,J)** *N*=3, **(C)** *N*=5, **(D,F)** *N*=8. **(K)** *N*=2. Representative blot.

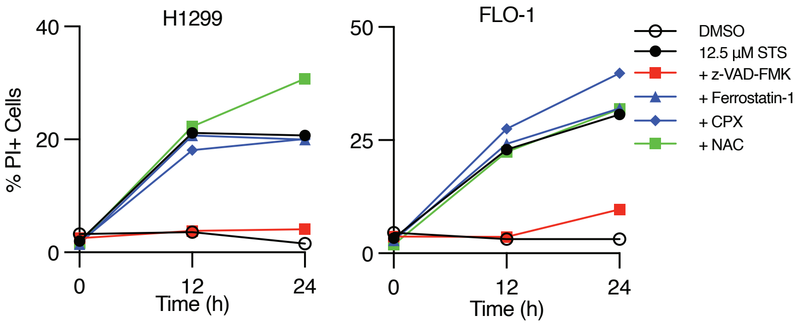

Figure S3. Relating to Figure 3 Eprenetapopt triggers ferroptosis
Cell death (% PI positive) induced by staurosporine (STS, 12.5 µM) with or without apoptosis (zVAD-FMK, 50 µM) or ferroptosis inhibitors (Fer-1, 12.5 µM; CPX, 6.25 µM; NAC, 2.5 mM) in H1299 and FLO-1 cells over 24 h. *N*=1.

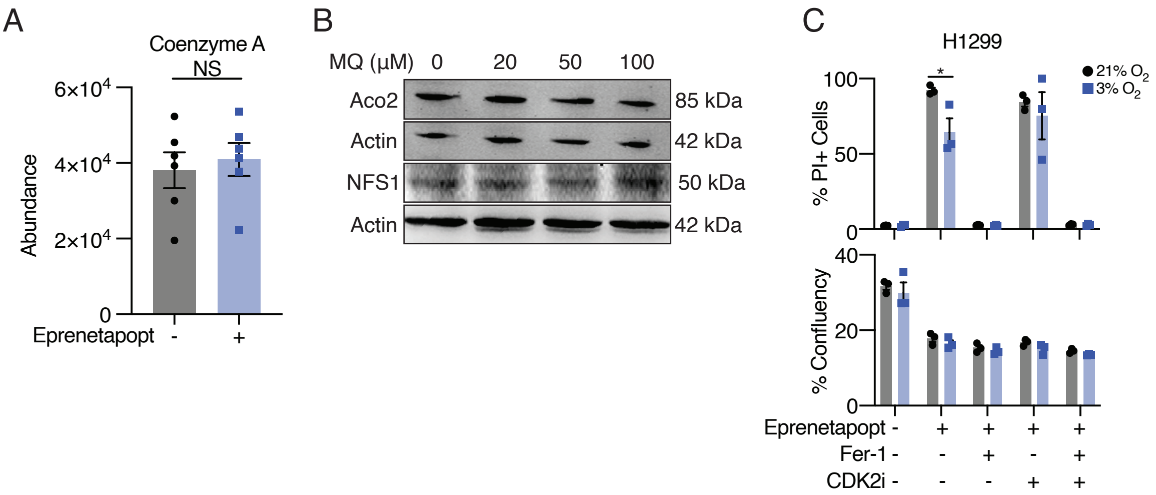

Figure S4. Relating to Figure 4 Eprenetapopt inhibits NFS1 cysteine desulfurase activity

(A) Absolute abundance of coenzyme A in OACM5.1 cells following treatment with 50 μM eprenetapopt from **Figure 1H**. **(B)** Immunoblot of Aco2 and NFS1 protein levels following 24 h exposure with MQ at indicated doses in 293T cells. **(C)** % cell death and % cell confluency following treatment for 24 h with 50 μM eprenetapopt with or without 12.5 μM Fer-1 and/or 625 nM CDK2 inhibitor (SNS-032) in 21% O_2 (black bars) or 3% O_2 (blue bars) in H1299. Two-tailed t-test **(C)**. * $P < 0.05$. Error bars = SEM. **(A)** *N*=6. **(B)** *N*=2, representative blots shown. **(C)** *N*=3.

SUPPLEMENTAL TABLES AND DATASETS

Table S1 List of oligonucleotides used for CRISPR sgRNA guides

Gene	Forward (5'-3')	Reverse (5'-3')
GCLM sgRNA_1	CACCGAATCAACCCAGATTTGGTCA	AAACTGACCAAATCTGGGTTGATTC
GCLM sgRNA_2	CACCGACTAGAAGTGCAGTTGACAT	AAACATGTCAACTGCACTTCTAGTC
SLC7A11 sgRNA_1	CACCGAAGGGCGTGCTCCAGAACAC	AAACGTGTTCTGGAGCACGCCCTTC
SLC7A11 sgRNA_2	CACCGTGAGCTTGATCGCAAGTTCA	AAACTGAACTTGCGATCAAGCTCAC
ESD sgRNA_1	CACCGAATGTACTCTTATGTCACAG	AAACCTGTGACATAAGAGTACATTC
ESD sgRNA_2	CACCGCCTGAGAGCCAATACAGTGC	AAACGCACTGTATTGGCTCTCAGGC
SHMT2 sgRNA_1	CACCGCTACTCACAAGACTCTTCGA	AAACTCGAAGAGTCTTGTGAGTAGC
SHMT2 sgRNA_2	CACCGTCATGCGGGCGTAGTCAATG	AAACCATTGACTACGCCCGCATGAC
MTHFD1L sgRNA_1	CACCGAGGACAAACTCCAAAAGCTG	AAACCAGCTTTTGGAGTTTGTCCTC
MTHFD1L sgRNA_2	CACCGTCGACCCATCTACCATCACG	AAACCGTGATGGTAGATGGGTCGAC
GCLC activation sgRNA	CACCGACACGCCTCCTGAGCCCCCG	AAACCGGGGGCTCAGGAGGCGTGTC

Table S2 List of antibodies

Antibody	Origin	Clone	Dilution	Source
Anti-SLC7A11	Rabbit	D2M7A	1:1000	Cell Signaling Technology
Anti-SHMT2	Mouse	F-11	1:1000	Santa Cruz Biotechnology
Anti-MTHFD1L	Rabbit	D8T7L	1:1000	Cell Signaling Technology
Anti-Bax	Rat	49F9	1:10000	Gift from Prof. Grant Dewson (WEHI)
Anti-Bak	Rabbit	N/A	1:10000	Sigma-Aldrich
Anti-Bid	Rat	4B5	1:1000	Gift from Prof. Ruth Kluck (WEHI)
Anti-Caspase-3	Rabbit	D3R6Y	1:1000	Cell Signaling Technology
Anti-Caspase-7	Rabbit	D2Q3L	1:1000	Cell Signaling Technology
Anti-NFS1	Rabbit	N/A	1:1000	Sigma-Aldrich
Anti-Aco2	Rabbit	N/A	1:4000	Sigma-Aldrich
Anti-Actin	Mouse	AC-74	1:8000	Sigma-Aldrich
Anti-Glutathione	Mouse	D8	1:1000	Abcam
Anti-Vinculin-HRP	Mouse	7F9	1:2000	Santa Cruz Biotechnology
Anti-GAPDH-HRP	Rabbit	14C10	1:10000	Cell Signaling Technology
Swine anti-rabbit	Swine	P0217	1:7000	Dako
Goat anti-mouse	Goat	P0447	1:7000	Dako
Goat anti-rat	Goat	3010	1:5000	Southern Biotech

Table S3 List of synthetic guides from Synthego for combinatorial CRISPR-editing in Mc38 cells

Gene	ID	Sequence
Casp3	2101	CATGCAGAAAGACCATACAT
Casp3	2103	AACCTCAGAGAGACATTCAT
Casp7	2107	GATATGCTTTAGGCATGCCG
Casp7	2108	TCCATGCGGTACAGATAAGT
Bid	1573	CCACAACATCCAGCCCACAC
Bid	1575	GCCAGCCGCTCCTTCAACCA
Bax	1429	GGACACGGACTCCCCCCGAG
Bax	1430	GTTTCATCCAGGATCGAGCA
Bak1	1410	GGAACTCTGTGTCGTAGCGC
Bak1	1412	GCAGGAGGCTCTTACCAGAA

Table S4 List of primers

Gene	Forward (5'-3')	Reverse (5'-3')
GCLC	CAGGACAGTTCTTAGATGCTGC	TTTGGGCCACACATAAGAAAGC
GAPDH	GGTGTGAACCATGAGAAG	CCACAGTTTCCCGGAG
ACTB	AGAAAATCTGGCACCACACC	GGGGTGTTGAAGGTCTCAAA

Dataset S1. Source data from Figure 1D and 1F. List of genes and MAGeCK scores for CRISPRko "drop-out" screen (average of two independent screens), CRISPRa "enrichment" screen (one screen) and DepMap gene dependency Fischer's z-transformed Pearson's correlation strengths.

Movie S1. H1299 cells die from 50 µM eprenetapopt treatment over 24 h. Here, we present a time-lapse video of cells with images taken every 5 min at 6 frames per second, therefore each second represents 1 h in real time, starting immediately after dosing with eprenetapopt. Images were acquired on IncuCyte FLR (Essen BioSciences).

Movie S2 A-D. Time-lapse videos of H1299 cells plated at low (2,500 cells/well, **A, B**) or high density (20,000 cells/well, **C, D**) and treated with 50 µM eprenetapopt (**B, D**) or vehicle (**A**, **C**) in the presence of propidium iodide (red). Images were taken every 15 min over 24 h and are shown at 4 frames per second, therefore each second represents 1 h in real time, starting immediately after dosing with eprenetapopt. Images were acquired on an IncuCyte FLR and phase contrast and red-fluorescent channels were overlaid.
